# Comparison of the macro and microstructure of sleep in a sample of sleep clinic hypersomnia cases

**DOI:** 10.1016/j.nbscr.2019.02.001

**Published:** 2019-02-14

**Authors:** Alyssa Cairns, Richard Bogan

**Affiliations:** a SleepMed, Inc., Columbia, SC, United States; b The University of South Carolina Medical School, Columbia, SC, United States; c The Medical University of South Carolina, Charleston, SC, United States; d Bogan Sleep Consultants, LLC, Columbia, SC, United States

**Keywords:** Spectral analysis, Signal processing, Spectrum, MSLT, Sensitivity, SOREMP

## Abstract

The purpose of this study was to elucidate the differentiating or grouping EEG characteristics in various hypersomnias (type 1 and type 2 narcolepsy (N-1 and N-2) and idiopathic hypersomnia (IH) compared to an age-matched snoring reference group (SR). Polysomnogram sleep EEG was decomposed into a 4-frequency state model. The IH group had higher sleep efficiency (SE; 92.3% vs. 85.8%; sp < 0.05), lower WASO (IH = 35.4 vs. N-1 = 65.5 min; p < 0.01), but similar (i.e. high) arousal indices as N-1 (~33/h). N-1 and N-2 had earlier REM latency than IH and SR (N-1 = 64.8, N-2 = 76.3 vs. IH/SR = 118 min, p < 0.05). N-1 and N-2 showed an increase in MF1 segments (characteristic of stage 1 and REM) across the night as well as distinct oscillations every 2 h, but MF1 segment timing was advanced by 30 min compared to the SR group (p < 0.05). This suggests the presence of circadian organization to sleep that is timed earlier or of increased pressure and/or lability. MF1 demonstrated a mixed phenotype in IH, with an early 1^st^ oscillation (like N-1 and N-2), 2^nd^ oscillation that overlapped with the SR group, and a surge prior to wake (higher than all groups). This phenotype may reflect a heterogeneous group of individuals, with some having more narcolepsy-like characteristics (i.e. REM) than others. LF domain (delta surrogate) was enhanced in IH and N-1 and more rapidly dissipated compared to N-2 and SR (p < 0.05). This suggests an intact homeostatic sleep pattern that is of higher need/reduced efficiency whereas rapid dissipation may be an underlying mechanism for sleep disruption.

## Highlights

• Low frequency sleep (delta surrogate) was enhanced in Idiopathic Hypersomnia and Type 1 Narcolepsy and rapidly dissipated across the sleep period.
• Type 1 and 2 Narcoleptics demonstrated a mixed frequency 1 phenotype (REM surrogate) consistent with intact circadian control and advanced timing.
• Idiopathic hypersomnia was characterized by a variable mixed frequency 1 phenotype (REM surrogate), suggesting some with more “narcolepsy-like” REM characteristics than others.

## 1 Introduction

Central nervous system (CNS) hypersomnias are characterized by profound sleepiness in the absence of other explanatory factors, such as behavioral sleep restriction, circadian rhythm misalignment, or other medical/psychological disorders (AASM, 2014). Included in this general category are narcolepsy and idiopathic hypersomnia. Narcolepsy is diagnostically partitioned into type 1 (N-1) and type 2 (N-2), with N-1 characterized by the presence of cataplexy or low CSF hypocretin and N-2 being characterized by the absence of cataplexy and/or normal hypocretin levels. Type 1 narcolepsy is believed to be caused by auto-immune mediated destruction of neuropeptide hypocretin neurons, which function to promote wakefulness, suppress rapid eye movement (REM) sleep, and provide state stability (Nishino et al., 2000). Consequently, patients with N-1 often complain of (in addition to cataplexy) disrupted sleep/wake continuity and often display abnormal sleep architecture upon laboratory study. For example, several studies have shown that, compared to controls, N-1 is associated with reduced sleep efficiency, increased wake after sleep onset (WASO), high arousal indices, frequent sleep stage transitions, increased time in “light” sleep (Sorensen et al., 2013, Pizza et al., 2015, Mukai et al., 2003, Khatami et al., 2008, Jiménez-Correa et al., 2009, Frauscher et al., 2011; Roth et al., 2013) and abnormal REM sequencing (Liu et al., 2015, Drakatos et al., 2016). It is also well-documented that N-1 patients have a shortened latency to nocturnal REM, now known to be statistically specific for low or absent hypocretin (Andlauer et al., 2013, Reiter et al., 2015; Cairns and Bogan, 2015).

From an etiological standpoint, N-2 is less well-understood than N-1 because cataplexy is absent, hypocretin levels are *ususually* normal (Andlauer et al., 2012), and patients less-frequently present with hypnogogic/pompic hallucinations and sleep paralysis (Khan and Trotti, 2015). In the absence of a reliable biomarker, the diagnosis of N-2 requires supportive data from the multiple sleep latency test (MSLT) and nocturnal polysomnogram (PSG). A test is considered supportive of narcolepsy if the patient has an MSLT mean sleep latency (MSL) of </=8 min and >/= 2 REM onsets between the PSG and MSLT (AASM, 2014). The diagnosis of IH is assigned when the MSLT reveals a MSL </= 8 min and 1 or fewer REM onsets on the PSG/MSLT.

The appropriateness and utility of the MSLT to diagnose hypersomnia and/or reliably distinguish between IH and N-2 has been the subject of much debate (Khan and Trotti, 2015, Baumann et al., 2014) because of methodological limitations and demographic factors that challenge the test’s interpretation (Johns, 2000). These include, but are not limited to, age-related attenuation in REM, medication use, circadian timing, and habitual sleep schedule (Baumann et al., 2014; Chakravorty and Rye, 2003). Additionally, there are a variety of environmental stimuli (light, sound, etc.) that can interrupt one’s ability to fall and stay asleep (and thus have REM) in a novel laboratory environment. All of the aforementioned factors result in low test-retest reliability of the MSLT, with only approximately 30–47% of individuals retaining their first diagnosis (either N-2 or IH) when repeatedly examined (Trotti et al., 2013, Ruoff et al., 2018, Lopez et al., 2017).

Considering these methodological concerns and the sheer need to better understand the underlying mechanisms and discerning characteristics of the central hypersomnias, there is increased interest in quantitative analysis (e.g. spectral analysis) of the nocturnal sleep period. Quantitative analyses elucidate high-resolution temporal changes in electroencephalogram (EEG) frequency population that are not adequately reflected using traditional scoring methods (Achermann, 2009). Existing literature on narcolepsy using quantitative analyses suggests impairments in both slow wave activity (SWA) and REM sleep processes including altered REM sequencing (Liu et al., 2015, Drakatos et al., 2016), attenuated circadian organization of REM (Mukai et al., 2003), and potentially hyper-sensitive homeostatic regulation of SWA (Khatami et al., 2008, Khatami et al., 2007, Yun et al., 2017). However, the majority of research has been conducted on narcolepsy with confirmed low or absent hypocretin, and very little is known about the regulation of nocturnal sleep in other hypersomnias, including N-2 and IH. Thus, the purpose of this study is to evaluate the characteristics of the nocturnal PSG using advanced signal processing to potentially elucidate the differentiating (or grouping) characteristics between the hypersomnias compared to an age-matched snoring reference group (SR).

## 2 Methods

Data for this study were retrieved from SleepMed’s repository of deidentified sleep studies conducted at various sleep disorders clinics in the United States between 2005 and 2015. SleepMed is a large network of sleep diagnostic facilities distributed across the United States. Data were signal processed (expanded below), technologist-scored on a 30-second epoch by epoch basis, and physician interpreted. The study protocol was approved by Schulman Associates institutional review board (IRB) for the protection of human subjects.

### 2.1 Measures

This study utilized information acquired from the patient’s scored and interpreted PSG as well as data acquired from the patient’s medical intake form. The intake form is a self-reported measure which inquiries about basic demographic information, previous diagnoses, current medications, sleep/wake habits, sleep/wake complaints, and symptoms of a variety sleep disorders. Sleepiness was assessed with the Epworth Sleepiness Scale (ESS; Johns, 1991).

### 2.2 Polysomnogram data acquisition and scoring

Data were acquired using a variety of native sleep systems from various sleep disorder laboratories across the United States using traditional electrode placement and preparation as per the AASM technical guidelines (Iber et al., 2007). Native data were exported to European data format and imported into an automated signal processing software (Pittman et al., 2004). Studies were visually graded to assess fitness for study inclusion; those deemed prohibitively artefactual due to electrical interference, pulse, sweat, or respiration artifact, or poor ground/reference placement were excluded. Prior to processing, raw tracings were conditioned with a 50/60 Hz notch and 0.5–30 Hz band pass filter. Feature extraction was employed to remove electrooculogram (EOG) and electrocardiogram (ECG) artifacts. Epochs with significant signal degradation (e.g. movement artifact) were removed from analyses. Morpheus^TM^ automated signal processing was performed using the C4/A1 derivation. Morpheus^TM^ decomposes EEG data into a 4-frequency state model using adaptive segmentation with fuzzy clustering and feature extraction (Pittman et al., 2004). The 4 frequency states include high frequency (HF), low-frequency (LF), and mixed frequency with high or low energy (MF1 and MF2, respectively). Membership in the HF domain is typical during wakefulness and includes both alpha (8–12 hz) and beta (13–30 hz) EEG bands, whereas LF is characteristic to delta bands seen in N3 sleep (0.5–2 hz). MF2 is characteristic of spindles and k-complexes observed in N2 sleep and MF1 is characteristic of both stage 1 and REM and typically includes waveforms in the theta range (3–7 hz). The EEG sleep stage scoring algorithm has shown good agreement compared to manual scoring of sleep staging using AASM criteria (k = 0.61–0.67) and fair agreement for REM (intraclass correlation coefficient; ICC = 0.72–0.76; Pittman et al., 2004). After Morpheus processing, registered sleep technologists reviewed data on an epoch-by-epoch basis for accuracy. Special attention is given to stage transitions and REM start/end.

### 2.3 Sample selection

Approximately 45 records from each diagnostic group were randomly selected from a deidentified database and reviewed for medical history and completeness of data. Patients with N-1, N-2, and IH were first identified based on the final diagnosis of the physicians interpretation reports and corroborated with MSLT results (i.e. narcolepsy = MSL</=8 min and >/=2 REMs and IH = </=1 REMs with MSL</=8 min; AASM, 2014). By definition, patients with N-1 (or narcolepsy with cataplexy, depending on the year of the diagnosis) had physician-documented cataplexy as per emotion-induced [typically positive] muscle weakness, facial/eyelid drooping, knees buckling, etc. Of note, 1 subject was originally categorized as IH (due to having only 1 MSLT REM onset and a MSL<8 min), however, they were subsequently re-assigned as N-2 due to the presence of a PSG short onset REM period (REM latency<15 min; AASM, 2014).

This study utilized a snoring reference group (hereafter referred to as SR) to compare the structure of sleep in those with vs. without a hypersomnia condition. These cases, although not “healthy” controls, were selected for mild presenting symptoms and no obvious sleep pathology on the nocturnal sleep study. That is, all SR cases demonstrated low-moderate Epworth scores (ESS < 10) and all demonstrated “normal” (per the physician) PSG structure and no sleep pathology (e.g. sleep apnea syndrome, bruxism, limb movements, etc.). Snoring reference cases were excluded if the patient ever had an MSLT (indicating suspicion for hypersomnia) or if they demonstrated a PSG SOREMP the night of their nocturnal sleep study.

### 2.4 Study inclusion and quality assurance

After general inclusion were satisfied, a thorough chart review was conducted to assess each subject’s medications, habitual sleep timing/duration, and sleep/wake symptoms. Sleep timing and duration was assessed via a single-item measure on the medical intake questionnaire (“*what is your typical bedtime and wake time on weekdays and weekends*”). Although actigraphy data were not available to confirm sleep habits prior to the sleep study (which can be particularly important to differentially diagnose N-2 and IH), to crudely rule out behavioral sleep restriction and circadian misalignment, subjects were excluded if they reported a sleep period < 8 h or if they reported a bedtime later than 1:00 a.m. Subjects were also excluded if they reported shift or night work, if they were using one or more psychotropic compound within 2 weeks of the sleep study (e.g. antidepressant, antipsychotic, sedative-hypnotic, anxiolytic, or stimulant), or if they underwent a partial or full titration. Because sleep apnea can be observed in Narcolepsy (Sansa et al., 2010) mild-moderate OSA was not an exclusion factor for the Narcolepsy groups. Mild-moderate OSA was, however, exclusionary for the IH and SR groups. Limb movements and PLMs were also not considered exclusion factors, with exception to the SR group. Lastly, raw data were graded for signal processing adequacy as per criteria in Section 2.2. In sum, these criteria yielded a final sample size of 103, with between 24-300 subjects in each group (Table 1).

**Table 1:** Demographics.

	**N-1**	**N-2**	**IH**	**Snoring Reference**	**Statistics**^**1**^
**N ( = 103)**	24	30	25	24	
					
**Anthropomorphics**					
BMI (kg/m^2^)	31.7 +/- 6.1	30.2 +/- 7.4	24.9 +/- 3.7	29.7 +/- 6.0	**F**_**(3,97)**_= 6.2; p = 0.001; *IH < all (p < 0.05)
Age [range]	35.2 +/- 14.6 (14–73)	35.9 +/- 12.1 (17–71)	36.4 +/- 13.4 (12–67)	34.6 +/- 11.7 (17–58)	p = 0.96
Sex (%Female)	58%	60%	60%	38%	p = 0.29
Race (%Black) ^2^	59%	36%	4%	22%	**Χ**^**2**^_**(3,96)**_**= 15.1; p = 0.002**
Diagnosed Depression	23%	10%	18%	17%	p = 0.42
					
**Sleepiness and Narcolepsy Symptoms**					
ESS ^3^	18.2 +/- 4.5	17.5 +/- 4.2	15.1 +/- 4.7	6.7 +/- 3.6	**F**_**(3,100)**_= 37.5; p < 0.001; *SR < all (p < 0.05)
%ESS>12	83%	90%	72%	0%	**Χ**^**2**^_**(3,100)**_**= 58.3; p < 0.001**
%ESS>16	74%	70%	48%	0%	**Χ**^**2**^_**(3,100)**_**= 58.3; p < 0.001**
Sleep Paralysis ^5^	31%	9%	6%	4%	p = 0.08
Hypnogogic Hallucinations ^6^	54%	23%	13%	8%	**X**^**2**^_**3,75**_**= 10.8; p = 0.01**
					
**Sleep Habits**^**7**^					
Weekday Bedtime (hr:min)	22:36 +/- 1:18	22:24 +/- 0:46	22:20 +/- 0:59	22:37 +/- 1:15	p = 0.72
Weekday Rise time (hr:min)	7:15 +/- 1:29	6:50 +/- 1:19	6:32 +/- 1:35	6:34 +/- 1:59	p = 0.40
Weekday Sleep Period (hr)	8.7 +/- 1.5	8.4 +/- 1.5	8.2 +/- 1.1	7.8 +/- 1.3	p = 0.15

^1^Bolded analyses = statistically significant (p < 0.05); X^2^ analyses for categorical variables; ANOVA for continuous variables; N1 = type 1 narcolepsy, N-2 = type 2 narcolepsy, IH = idiopathic hypersomnia, SRC = snoring reference group, all = all groups; ^2^ a small number of Hispanic and Asians were present (n = 7), but were excluded from categorical analyses; ^3^ ESS = Epworth Sleepiness Scale; ^5^ “when falling asleep, how often do you feel unable to move or paralyzed? [sometimes or more]”; ^6^ when falling asleep, how often do you experience vivid, dreamlike scenes or hallucinations even though you are awake? [sometimes or more]”; ^7^ derived from medical intake questionnaire (“*what is your typical bedtime and wake time*?”), sleep period = difference between bedtime and rise time; * significant pairwise comparison.

### 2.5 Statistical analyses

Analyses were performed using SPSS 23.0 (SPSS Inc., Chicago, IL). For continuous variables, descriptive analyses were completed to analyze the shape, central tendency, and dispersion to ensure parametric testing appropriateness. Distributions with statistical outliers were windsorized. Due to the nature of repeated-measures analyses needing equal across-time iterations, the first 7 h from lights out (i.e. the shortest sleep period length of the sample) was selected for advanced signal processing. This 7-h period started at a mean lights out of 22:12 +/ 32 min and ended at an ‘artificial’ lights on of 5:12. Each subject’s 7-h PSG period was subsequently partitioned into 5-min time bins (n = 82 repeated time measures). Electroencephalographic segment population over time was assessed with repeated measures analysis of variance (ANOVA) with Bonferroni post-hoc comparisons (factor = group [n = 4] with repeated measure = time [n = 82]). Significant time by group interactions were further assessed with univariate ANOVA with Bonferroni pairwise comparisons at each time bin. Group differences in demographics and PSG macroarchitecture were assessed with either ANOVA or Chi Square analysis depending on the nature of data. All comparisons were two-tailed and significance was set at the 0.05 level.

## 3 Results

As expected, N-1 cases were remarkably sleepy (74% with ESS >16) and reported the highest rate of hypnogogic hallucinations (p < 0.01; Table 2). Also, N-1 patients were noted to be disproportionately Black and numerically reported the highest prevalence of sleep paralysis (although statistically marginal; p = 0.08). Type 2 Narcolepsy cases were also remarkably sleepy (70% with ESS >16), but were less-likely than those with N-1 to report hypnogogic hallucinations (23% vs. 54%; X^2^_1,34_ = 4.0; p = 0.04). Although current diagnostic criteria do not differentiate IH based on habitual sleep duration (AASM, 2014), because these data were archival, IH studies done prior to 2014 many times received a diagnosis with ‘IH *without* long sleep time’. Of note, the majority of the IH sample self-reported a fairly ‘typical’ habitual sleep period length; only 3 IH cases reported a sleep duration of 9 h or longer (Table 1). On average, the IH group reported a high degree of sleepiness (mean ESS was more than twice that of the SR), but fewer reported extreme sleepiness (ESS>16) than either narcolepsy group (48% vs. 73% [combined narcolepsy rate] X^2^_1,76_ = 4.4; p = 0.03; Table 1). The IH group was also noted to be disproportionately White (92%).

**Table 2:** Polysomnogram macroarchitecure.

	**N-1**	**N-2**	**IH**	**Snoring Reference**	**Statistics**^**1**^
**N = 103**	24	30	25	24	
					
**Endpoint Data**					
PSG Total Sleep Time	401.2 +/- 75.2	398.0 +/- 56.1	408.1 +/- 31.8	390.9 +/- 19.1	p = 0.69
AHI 4%	2.4 +/- 3.0	1.9 +/- 1.8	1.0 +/- 0.8	1.7 +/- 1.4	p = 0.10
% AHI > 5	13%	7%	0%	0%	p = 0.11
RDI	3.9 +/- 3.6	4.0 +/- 2.8	2.5 +/- 1.6	3.2 +/- 2.3	p = 0.13
Initial Sleep Latency (min)	14.0 +/- 17.7	19.1 +/- 21.0	14.2 +/- 14.0	20.3 +/- 16.7	p = 0.46
Sleep Efficiency (%)	85.8 +/- 12.2	90.2 +/- 8.8	92.3 +/- 4.6	91.6 +/- 4.4	**F**_**(3,99)**_= 3.2; p = 0.03; *IH > N-1 (p = 0.04)
WASO (min)	65.5 +/- 53.0	44.2 +/- 38.1	35.4 +/- 21.3	37.0 +/- 20.4	**F**_**(3,99)**_= 3.6; p = 0.02; *N-1 > SR (p = 0.04), N-1 > IH (p = 0.02)
Arousal Index (#/hr)	32.8 +/- 19.1	25.3 +/- 11.8	33.1 +/- 24.6	19.6 +/- 7.8	**F**_**(3,99)**_= 3.3; p = 0.02; *IH > SR (p = 0.04), N-1 > SR (p = 0.04)
PLM Index (#/hr)	6.6 +/- 13.1	4.6 +/- 8.5	2.8 +/- 8.1	0.9 +/- 1.7	p = 0.14
					
**Sleep Architecture**					
%N1	8.1 +/- 6.1	8.1 +/- 6.4	7.9 +/- 5.0	6.9 +/- 4.5	p = 0.86
%N2	52.5 +/- 7.2	49.4 +/- 7.8	54.6 +/- 8.4	53.3 +/- 8.7	p = 0.10
%N3	18.1 +/- 6.7	17.9 +/- 4.8	16.0 +/- 6.3	18.8 +/- 4.6	p = 0.34
%REM	21.0 +/- 8.0	24.5 +/- 7.0	21.5 +/- 6.1	21.0 +/- 5.4	p = 0.21
					
**REM Data**					
REM Latency (RL; min)	64.8 +/- 62.0^^^	76.3 +/- 49.2	118.4 +/- 55.2	118.4 +/- 57.9	**F**_**(3,98)**_= 5.7; p = 0.001; *N-1 < SR (p < 0.01), N-2 < SR (p = 0.04)
% SOREMP (RL < 15 min)	29%^^^	17%	0%^+^	0%	**X**^**2**^_**3,103**_**= 14.3; p = 0.01**

^1^Bolded analyses = statistically significant (p < 0.05); X^2^ analyses for categorical variables; ANOVA for continuous variables; N1 = type 1 narcolepsy, N-2 = type 2 narcolepsy, IH = idiopathic hypersomnia, SR = snoring reference group, all = all groups; AHI = apnea hypopnea index = the average number of apneas and hypopneas (4% desaturation) per hour of sleep; RDI = respiratory disturbance index = the average number of apneas, hypopneas (4%), and flow limited events that either terminate in an EEG arousal or a 3% desaturation per hour of sleep; PLM = periodic limb movement; ^^^ One subject had no scored REM [analyzed as a missing value], but omnibus result did not change when excluding this subject; ^+^ 1 IH subject was moved to the N-2 category due to having a PSG SOREMP and 1 MSLT REM onset; * significant pairwise comparison.

### 3.1 Macroarchitecture

Patients with N-1 were similar to those with N-2 regarding most of the macroarchitecture values, including arousal index, WASO, and sleep efficiency, but those with N-1 had a greater prevalence of a PSG SOREMP than those with N-2 (29% vs. 17%, p < 0.05). However, the N-1 group had notably variable PSG TST, sleep efficiency, WASO, and arousal indices (Table 2). Those in the IH group had lower WASO (IH = 35.4 vs. N-1 = 65.5 min; p < 0.01) and higher sleep efficiency (92.3% vs. 85.8%; p < .05) compared to those with N-1, but no differences were detected between the N-2 and IH. Interestingly, the IH group demonstrated high arousal indices (33.1 vs. 19.6/h in SR; p < 0.05), numerically similar to the N-1 group (32.8/h). Variability in AI was high for the IH group, however. As a group, those with IH demonstrated fairly ‘typical’ REM onset latencies, similar to the value seen in the SR group (118 min). No differences were found in sleep onset latency or sleep stage distribution of between any of the groups.

### 3.2 EEG segment analyses

**Fig. 1:**
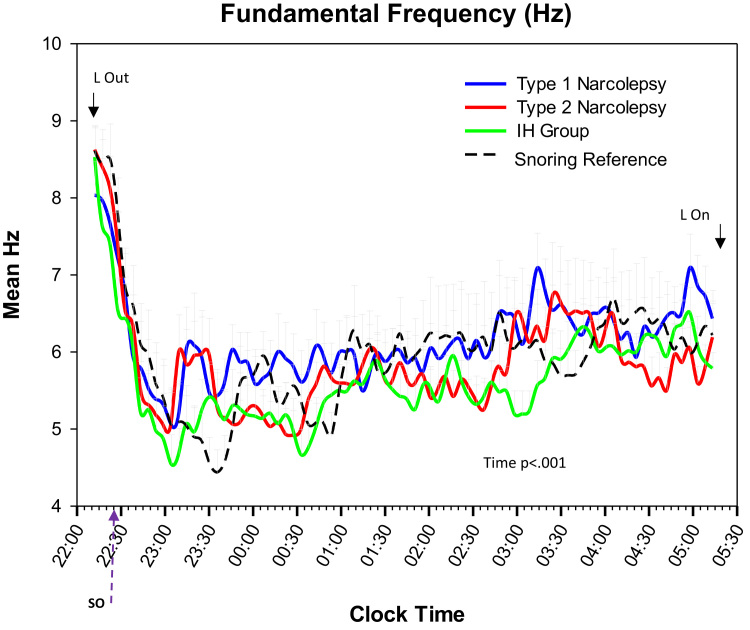
Fundamental Frequency (Hz) Across time. Fundamental frequency = mean EEG cycles per second (Hz). Each vertical tick on the X-axis represents a 5-min time bin of the PSG (PSG = the first 7 h from Lights out; mean of 22:12 +/− 32 min). SO = mean sleep onset for all groups (no statistical differences between groups). Trend lines represent spline-smoothed changes in mean fundamental frequency over time. Bars represent standard error bars (SEM). Mixed model ANOVA with Bonferroni post-hoc comparisons was used to assess group (N = 4) by time (N = 82 time increments) changes. Main effect of time present with no interaction or group effects.

**Fig. 2:**
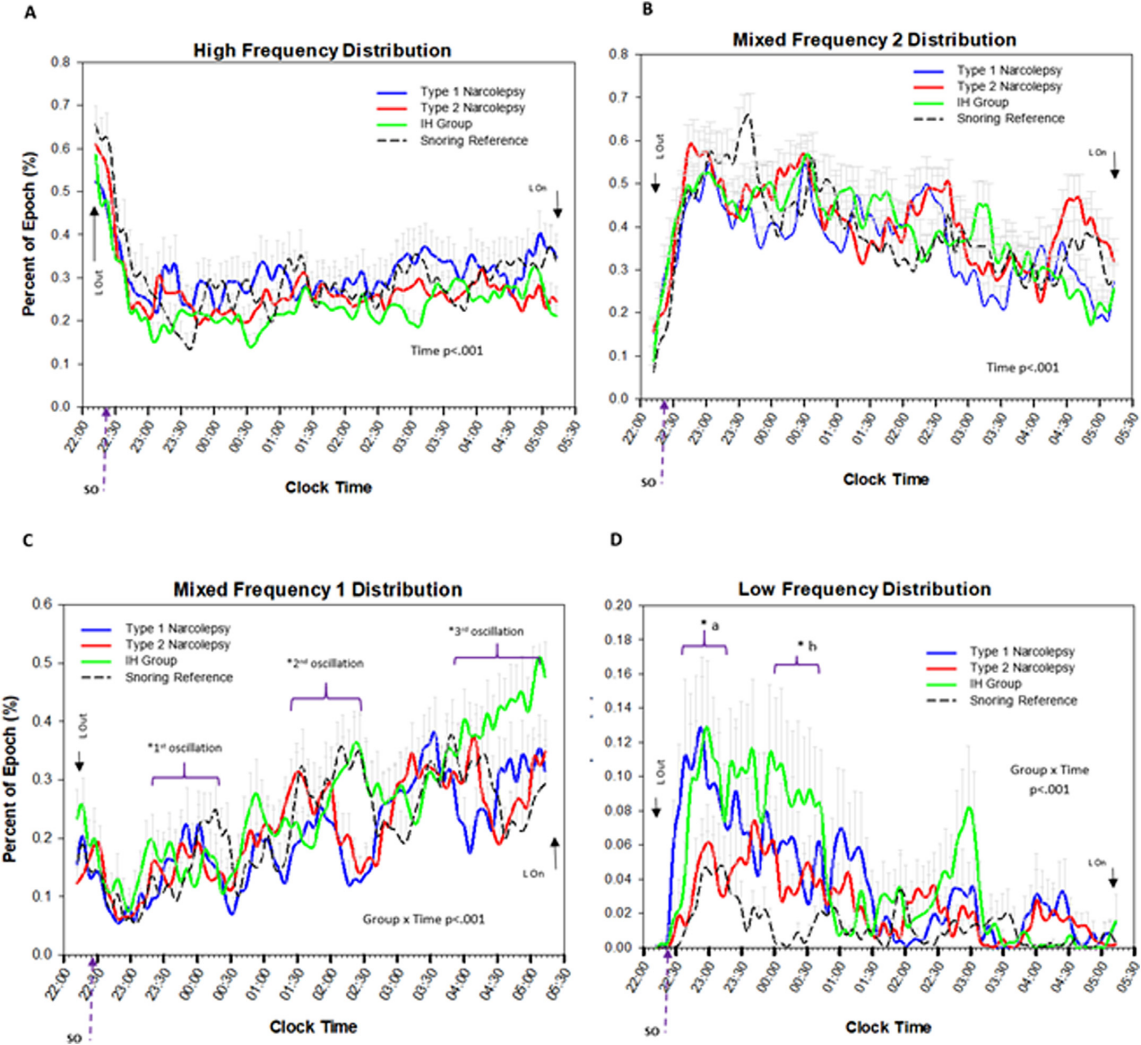
Time-related changes in percent of epoch comprised of each respective EEG frequency segment across time. HF = characteristic of wakefulness, MF2 = characteristic of stage 2, MF1 = characteristic of stage 1 and REM; LF = characteristics of stage 3. Each vertical tick on the X-axis represents a 5-minute time bin of the PSG (PSG = the first 7 h from Lights out; mean of 22:12 +/− 32 min). SO = mean sleep onset for all groups (no statistical differences between groups). Trend lines represent spline-smoothed changes in mean percent of time comprised of each frequency component over time. Bars represent standard error bars (SEM). Mixed model ANOVA with Bonferroni post-hoc comparison to assess group (N = 4) by time (N = 82 time increments) changes. 2 A&B-main effect of time observed, no interaction or group effects. 2C- group by time interaction (p < 0.001); 1^st^ oscillation- early in N-1, N-2 & IH compared to snoring controls p < .05), 2^nd^ oscillation- N-1 & N-2 early vs. IH & snoring reference (p < 0.05); 3^rd^ oscillation- N-1 & N-2 early vs. IH & snoring reference (p < 0.05). 2d- group by time interaction (p < 0.001); *a = N-1 & IH high LF domains than N-2 & snoring reference at acrophase (p < 0.05); *b = IH higher LF domains than all others (p < 0.05); precipitously decreasing at 1:00.

All groups similarly showed a linear decrease in mean fundamental frequency (mean cycles per second (Hz) at each 5-minute bin) as the night progressed, illustrating the decrease in mean Hz from wakefulness to sleep (F_81, 8019_ = 10.7; p < 0.001; Fig. 1). Likewise, all groups showed similar patterns of time-related decreases in high frequency (HF; F_81, 8019_ = 11.9; p < 0.001; Fig. 2A) and MF2 segments (F_81, 8019_ = 10.1; p < 0.001; Fig. 2B). Regarding MF1 segments, three notable observations were found. First, all groups showed an increase in the proportion of EEG comprised of MF1 segments as night progressed (main effect of time; F_81, 8019_ = 11.8; p < 0.001) with discernable oscillations occurring every 2 hours (Fig. 2C). Second, the timing of oscillations varied between groups (group by time interaction; F_243, 8019_ = 1.4; p < 0.001), with both narcolepsy groups exhibiting remarkable similarities in visual MF1 patterns, with each oscillation acrophase occurring 30 min earlier than SR (all oscillation pairwise comparisons p < 0.05; Fig. 2C). The IH group demonstrated a mixed MF1 phenotype. That is, as a group, they exhibited an early 1^st^ MF1 oscillation similar to narcolepsy groups (at 60 min, 80 min, and 110 min after sleep onset for IH, narcolepsy, and SR, respectively), but their 2^nd^ oscillation overlapped with the SR group (approximately 4 hours after sleep onset; p > 0.05), and they exhibited an increasing MF1 trend prior to lights on (~5:12 a.m.) compared to all other groups (pairwise comparisons p < 0.05). Although it appeared that there was an additional small oscillation between the 1^st^ and 2^nd^ oscillation, statistical comparisons were not significant (p = 0.30).

Low frequency segment analyses revealed a main effect of time (F_81, 8019_ = 6.8; p < 0.001) and group (F_3, 99_ = 3.9; p = 0.01; N-1, IH > SR, N-2; p < 0.05) as well as a group by time interaction (F_243, 8019_ = 1.3; p = 0.002; Fig. 2D). This suggests that all groups showed a dissipation of LF domains as the night progressed, but the time-course varied between the groups. Specifically, heighted LF domains were observed for the IH and N-1 groups compared to the N-2 and SR groups during the 1^st^ third of the PSG. At LF acrophase (38 min after lights out; ~22:50), 13% of EEG segments were comprised of LF domains in N-1 and IH groups compared to only 5% in SR and N-2 subjects (Fig. 2D). Low frequency domains remained relatively high in the IH group into the 2^nd^ hour of the sleep period (~22:30), compared to all other groups (9% vs. <5%; p < 0.05; Fig. 2D). In the IH group, LF domains precipitously degraded to < 1% at the 3^rd^ hour (~1:00), and no group differences in LF domains were observed thereafter. Because all groups had < 1% LF prior to lights on despite higher baselines in the IH and N-1 groups, we conclude that the rate of LF dissipation was more precipitous in both N-1 and IH groups compared to the N-2 and control groups.

## 4 Discussion

### 4.1 Differentiations between N-1 and N-2

Although we would have expected N-2 subjects to have lower sleepiness and better sleep continuity than those with N-1 (Pizza et al., 2015), as a group, statistical comparisons between the two groups revealed statistically similar sleep onset latencies, arousal indices, WASO, and sleep efficiencies. A few explanations are possible for this finding. First, and arguably most important, is the possibility of diagnostic misclassification within our N-1 group. That is, because CSF hypocretin values were not available in our sample (which is common in sleep clinic environments) and no standardized metric of cataplexy is routinely used, it is certainly possible that a portion of the N-1 group was not hypocretin-deficient afterall and thus were actually N-2 or another hypersomnia condition. The same applies for the N-2 group, that is, a portion of the N-2 group may have actually been hypocretin deficient. In fact, research has shown that approximately 24% of patients originally diagnosed as N-2 are indeed hypocretin deficient, and thus actually type 1 narcoleptic (Andlauer et al., 2012).

Alternatively, it cannot be ruled out that N-1 is simply a heterogeneous condition, with some individuals exhibiting high sleep efficiencies and low arousal indices, but perhaps high REM fragmentation, etc. The heterogeneity observed in this study is one of the most valuable findings because most of the data published to date are on hypocretin-deficient (vs. not) narcolepsy cases, which may be an entirely different phenotype altogether than what is naturally observed in a sleep clinic setting. Additional research is needed on the clusters of narcolepsy presentations in the sleep clinic setting. Nevertheless, it appears imperative to systematize the use of standardized and validated cataplexy measures (e.g. Leary et al., 2014) as well as non-invasive biomarker tests when available, such as HLA typing.

In addition to similarities in macroarchitecture, quantitative analysis of the PSG also revealed similarities in MF1 segments for both narcolepsy groups compared to others. Although MF1 is characteristic of both stage 1 and REM, the undulating and increasing nature of MF1 segments across the night typified the circadian pattern of REM, and thus, we interpret this finding to be mostly attributable to REM evolution across the night. Based on the assumption that MF1 oscillations reflected REM activity, we conclude that patients with narcolepsy (both types) exhibit ‘normal’ amounts of REM sleep (based on both micro and microstructure analyses) with robust circadian organization. This is contrary to what was found in a sample of 8 drug-naïve narcolepsy with cataplexy subjects, where they *did not* demonstrate increasing REM period durations over the course of the night (Mukai et al., 2003). Potential explanations for this difference include differing analytic techniques and increased statistical power, and supports Dantz and colleagues’ study of core body temperature (a measure of circadian timing) in narcolepsy under forced desynchrony conditions (Dantz et al., 1994). The finding that MF1 oscillations were advanced by approximately 30 min may be due to intrinsically earlier-timed REM, increased REM pressure, or an altered interaction of homeostatic and REM mechanisms.

Low frequency domain sleep, however, revealed different trends between the narcolepsy groups. That is, the composition of sleep in the first 3^rd^ of the night was disproportionately comprised of LF segments in N-1 with a precipitous degradation noted between the 1^st^ and 2^nd^ third of the night, whereas N-2 demonstrated a LF phenotype similar to the SR group (i.e. gradual onset and offset). This is similar to other reports of SWA dissipation in N-1 and may elucidate a potential mechanism for disrupted sleep continuity (Mukai et al., 2003; Khatami et. al., 2008; Khatami et. al., 2007). However, the aforementioned hypothesis does not explain the *overall* heightened LF domain activity observed in N-1. Although additional research is needed, one potential explanation for heightened LF activity is a homeostatic *response* to interrupted sleep continuity. However, it cannot go without recognition that elevated LF sleep seen in N-1 may be due to the nature of our control group, being that *they* may have had abnormally low LF domain sleep as a result of primary snoring.

### 4.2 Differentiations between Narcolepsy and IH

Very little empirical data exist on the characteristics of nocturnal sleep in IH compared to narcolepsy. Rather, most of the published literature highlights the de-facto differences in REM tendency on the MSLT and patient-facing symptoms such as the restorative quality of naps, sleep inertia, and length of sleep episodes, etc. (Khan and Trotti, 2015, Baumann et al., 2014, Mignot et al., 2006). Of the data that does exist on the characteristics of nocturnal sleep in IH, when compared to N-1, IH has been associated with increased sleep efficiency, longer REM onsets, and perhaps increased slow-wave activity (Anderson et al., 2007, Pizza et al., 2015). Findings from this study support these previous reports, however, our data also suggested that IH was associated with notably high arousal indices. Although this finding requires replication and further analyses (e.g. examination by symptoms of sleep drunkenness, etc.), high arousal indices may partially explain the non-restorative sleep in this group. High variability in arousals were noted, however. Like data from the narcolepsy groups, some individuals had ‘clusters’ of presentations (i.e. some with low arousals and well-consolidated sleep, some with more disrupted sleep).

Very little data is available on how N-2 quantitatively differentiates from IH. The most comprehensive evaluation of N-2 compared to IH conducted by Pizza and colleagues (Pizza et al., 2015) suggested that, other than REM latency (N-2 < IH), very little differed on the nocturnal PSG. Macrostructure findings from this study reinforce Pizza et al's findings. However, microarchitecture analyses of the IH group revealed shared *and* differentiating characteristics with *both* narcolepsy groups, however. That is, in this study, LF domain characteristics for IH patients clustered more with the N-1 group than N-2 group (i.e. elevated and rapid dissipation). Although these data require further examination, similar to the explanation in N-1 above, increased LF domain may be compensatory to and/or an underlying mechanism of sleep disruption (i.e. reduced sleep intensity).

It is also possible that this increased LF domain may explain some of the clinical features of “heavy sleep” reported by some IH patients. Of note, however, the finding that LF sleep dissipated to that observed in the snoring reference group and narcolepsy toward the end of the sleep period was contrary to prediction. We expected to see relatively elevated LF domain (a potential explanation for sleep drunkenness) the minutes prior to waking, but instead LF values dissipated to close to 0–1% prior to lights on. This may be due to the nature of our IH sample in that the majority were not “long sleepers” and data on sleep drunkenness was not available, so stratification could not be completed. It is also possible that we ‘missed’ meaningful changes in LF domains (and other EEG changes) toward the natural end of the sleep period because we artificially curtailed analyses at 7 h from lights out, where some individuals had up to 35 min of additional sleep. The finding that the IH group demonstrated a mixed MF1 phenotype suggests that this group may have been comprised of a mixture of individuals, perhaps with varied disease progression and some with more narcolepsy-like characteristics (i.e. REM) than others. The finding of elevated MF1 activity for the IH group during the last 90 min requires further investigation, but it may reflect a cluster of individuals with a longer circadian period or individuals with delayed circadian phase.

## 5 Limitations and future directions

This study had two main limitations (1) the retrospective nature of our dataset and (2) potential diagnostic overlap. Retrospective datasets are almost always less desirable than carefully-controlled prospective trials. Specific to this dataset was that limited information was available on cataplexy, sleep/wake habits, medications, and other comorbid psychiatric, sleep/wake, or other medical conditions. Importantly, although we excluded patients on psychotropic compounds, many medications (which were not excluded; e.g. beta blockers, thyroid hormone, diuretics, etc.) can meaningfully influence sleep and wakefulness. Potential diagnostic overlap was also a limitation for our study, but highlights important future directions for the assessment of hypersomnia in the clinical environment. Our data highlight the need for standardized, validated cataplexy instruments when assessing hypersomnia. Additionally, because CSF hypocretin is not routinely assessed in the clinical setting due to the cost and invasiveness of the procedure, more accessible mechanisms to assess this valuable biomarker are needed. Another main limitation to our study was the nature of our clinical control group. Although our snoring reference group were specifically selected for low AHIs/RDIs, modest levels of sleepiness, low sleep disturbance on the PSG, and otherwise ‘normal’ test results per the physician, it is certainly possible that snoring interrupted sleep at the microarchitecture level. This, in itself, is clinically meaningful and should be separately explored. Additionally, a subset of our snoring reference group (as well as our hypersomnia groups) had diagnosed depression, a condition that may endogenously alter REM tendency and amount (Palagini et al., 2013). Another limitation to this study was regarding variance in PSG study duration. Future signal processing studies should observe the ‘natural’ transition to sleep onset instead of that which is often artificially imposed in a sleep lab environment.

Lastly, and very importantly, these data question the utility of the MSLT to compartmentalize various hypersomnias in heterogeneous sleep clinic populations. That is, although the design of this study used carefully-reviewed chart notes, a variety of quality assurance metrics, and empirical outcomes [MSLT] with physician diagnoses (all which should give us fair confidence in our ‘groups’), high variability within the hypersomnia categories suggested some phenotypic overlap, which may be attributable to limitations of the test itself (Trotti et al., 2013, Ruoff et al., 2018, Lopez et al., 2017, Johns, 2000) and/or a spectrum hypersomnia phenomenon. Despite these limitations, this study had a number of unique strengths. Namely, to the best of our knowledge, this is the first study to quantify the time-course of EEG frequencies on the nocturnal PSG for a variety of hypersomnia conditions, including IH, which is very under-explored. Also, our dataset represented carefully-selected clinical cases, thus conclusions have high external validity and “real world” application, and ultimately highlight the need for a better understanding of the underlying mechanisms of the hypersomnias.

## Financial support

This study was funded by Jazz Pharmaceuticals.

## Conflicts of interest

Drs. Bogan and Cairns have received research support from Jazz Pharmaceuticals and are employed by SleepMed, Inc. Dr. Bogan also serves on the speakers bureau for and is a consultant to Jazz Pharmaceuticals.
